# A Gecko-Inspired Soft Passive Gripper

**DOI:** 10.3390/biomimetics5020012

**Published:** 2020-03-25

**Authors:** Arthur Seibel, Mert Yıldız, Berkan Zorlubaş

**Affiliations:** Workgroup on System Technologies and Engineering Design Methodology, Hamburg University of Technology, 21073 Hamburg, Germany; mertyldz94@gmail.com (M.Y.); berkanzorlubas@gmail.com (B.Z.)

**Keywords:** soft robotics, fluidic elastomer actuators, soft passive gripper, gecko-inspired adhesive

## Abstract

This paper presents a soft passive gripper consisting of six fluidic soft bending actuators arranged in a star-shaped manner. The actuators are oriented such that, upon pressurization, they bend against gravity. Gripping is realized by a commercial tape with mushroom-shaped adhesive structures that is glued to the bottom patches of the gripper. In this way, the object is released by peeling away the actuators from the object’s surface. In contrast to active grippers, which require continuous pressurization during gripping and holding, the presented passive gripper only requires energy for the release process. However, due to its working principle, the gripper is restricted to only flat objects or objects with at least one flat surface.

## 1. Introduction

Recent advancements in soft robotics have brought a broader perspective to robot designs, and new opportunities have been born to overcome the limits of robots with rigid bodies that reduce their ability to interact with their environment [1,2,3,4,5,6]. Accordingly, soft grippers [7] are increasingly studied as they provide high compliance and versatility and are safe to interact with. Unlike rigid grippers, which require precise motion accuracy for safety, soft grippers mainly offer high operational flexibility. Materials soft grippers are made of, such as silicone rubbers, are nonresistant to compressive forces and can withstand enormous strain deformations, which is why soft grippers can gently conform to objects with any shape, without harming them.

A variety of actuation principles in soft grippers were investigated [7] and many studies employ the use of air pressurization where the gripper’s shape is changed by applying pressurized air. Here, the links of the grippers are typically designed as soft bending actuators.

Current soft grippers are typically designed based on an active gripping principle, which means that actuation is required to maintain gripping during the entire operation. Here, the gripper’s body is kept pressurized and bent until detachment is intended. However, the active gripping principle also has some potential drawbacks. High strain deformations of soft materials might develop fatigue under pressure. Furthermore, material wear-off can constrain the duration of the gripper’s operation. Another drawback is that the application range of link-based soft grippers is limited by the fact that the object to be gripped should fit to the region enclosed by the links of the gripper, which means that a feasible range of payload size is directly linked to the gripper’s size.

In this paper, we introduce a soft passive gripper consisting of six soft bending actuators arranged in a star shape. The actuators are oriented to bend upwards to reduce the initial contact with the object. At the bottom patches of the actuators, a commercial tape with mushroom-shaped adhesive structures [8] is applied, which enables adhesion. Upon pressurization, the bending actuators peel away from the object, thus releasing it. The idea is inspired by the peeling process of a gecko’s feet [9], which exhibit very high adhesion when in contact with a surface.

The presented gripper expands the range of existing soft grippers with gecko-inspired adhesives. For example, Song et al. [10,11] introduce a soft gripper consisting of gecko-inspired adhesives in a flexible, inflatable membrane which attaches to flat as well as nonflat 3D objects by initially applying a normal force. Upon inflation of the membrane, it is peeled off from the object’s surface and the object is released. In contrast, Hawkes et al. [12] developed a soft gripper utilizing gecko-inspired adhesives that conforms to convex objects using exclusively shear forces. A design combining a “classical” soft gripper with soft bending actuators and gecko-inspired adhesives is presented in [13]. Compared to these solutions, our gripper attaches completely passively to the object to be lifted as the normal force is induced from the weight of gripper. However, our solution is not capable of attaching to nonflat 3D objects, contrary to the previous studies. Furthermore, our study gives more insight into the dynamics of a very commonly used soft actuator [14] and proposes a design methodology which can be used in the future for the design and application of soft passive grippers.

## 2. Concept Development

**Figure 1:**
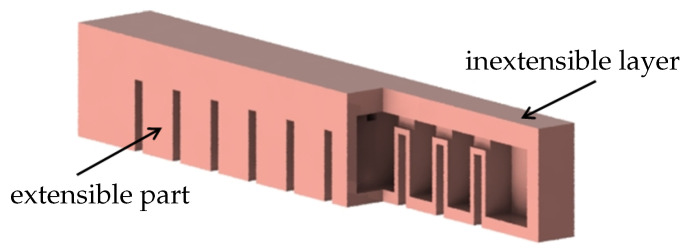
Partially cut fPN soft bending actuator.

The design concept of the passive gripper is based on the fast pneu-net (fPN) soft bending actuator [14]. This type of actuator is made of an inextensible top layer and an extensible, but bendable, bottom part, see Figure 1. The bending motion results from pneumatic actuation of the bottom part. In order to make bending easier, the bottom part is divided into multiple air chambers creating a repellent pushing effect towards the adjacent chamber walls. By placing an adhesive layer at the bottom of the actuator, a soft passive gripper can be created in that way.

**Figure 2:**
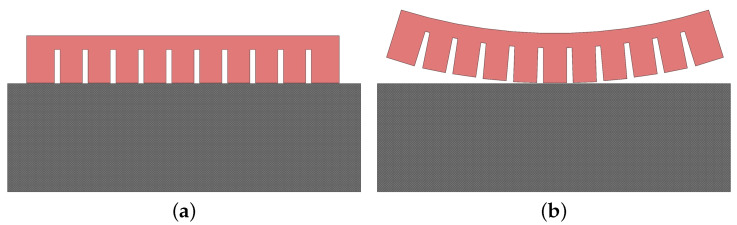
Attachment and detachment process of a soft bending actuator as soft gripper: (**a**) attached, uninflated; (**b**) detached, inflated.

Figure 2 shows a possible attachment (a) and detachment configuration (b) of an fPN bending actuator used as a soft gripper. When the actuator is flat, the object with flat surface is gripped by adhesion. However, when the actuator is inflated, it detaches from the surface and releases the object. Here, the repellent force between the air chambers creates a shear stress underneath the chambers, which, if large enough, causes the adhesive layer to detach.

**Figure 3:**
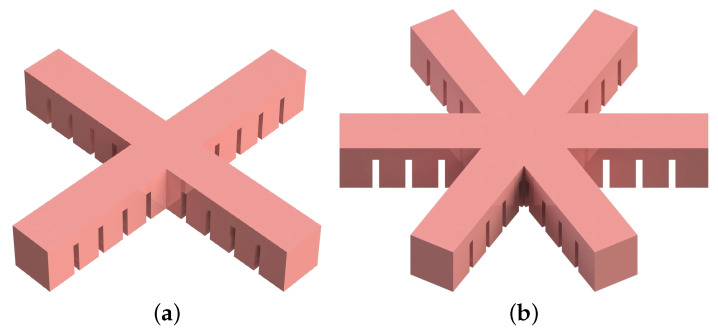
Variation of the number of soft bending actuator branches in the gripper: (**a**) four branches, (**b**) six branches.

As illustrated in Figure 3, the number of branches is also a parameter in the design of the soft gripper. It is directly related to the adhesion and grip force, which means that grippers with more branches can carry heavier loads but require a stronger pressurization.

**Figure 4:**
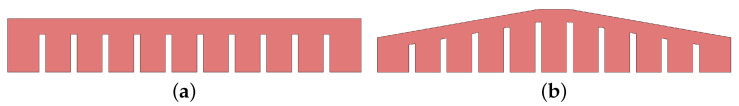
Variation of the shape of the soft bending actuator branches in the gripper: (**a**) flat, (**b**) sloped (in the displayed configuration, α is negative).

Another design variation can be made by giving branches a slope, as depicted in Figure 4. A slope in the main body of a soft bending actuator changes how bending creates a pulling force distribution at the adhesive layer. The required air pressure ($p_{\mathrm{air}}$) to overcome the adhesion ($\sigma_{\mathrm{adh}}$) between the gripper and the gripped object is estimated analytically in Appendix A. It is found that the relationship between $p_{\mathrm{air}}$ and $\sigma_{\mathrm{adh}}$ can be approximated by

(1) $$\frac{p_{\mathrm{air}}}{\sigma_{\mathrm{adh}}}\approx \frac{1}{\tan^{2}\left(\alpha \right)}=\cot^{2}\left(\alpha \right)\;,$$

where α describes the slope angle of the gripper. This is in analogy with the Kendall model [15] for adhesive tape peeling, as discussed in Appendix B and Appendix C.

**Figure 5:**
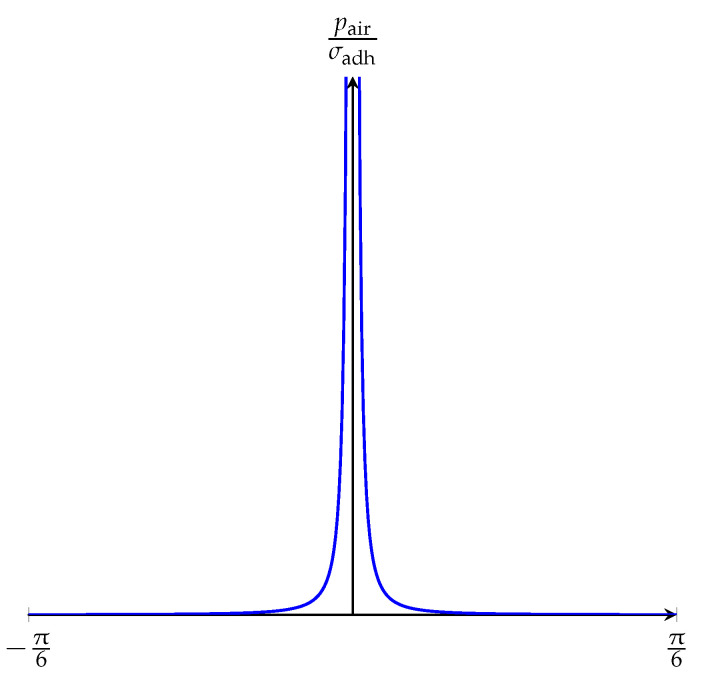
Graph of Equation (1).

The plot of Equation (1) is illustrated in Figure 5. We can see that $p_{\mathrm{air}}/\sigma_{\mathrm{adh}}$ goes to infinity as α goes to zero. In real-life applications, however, we do not expect the ratio of $p_{\mathrm{air}}$ to $\sigma_{\mathrm{adh}}$ to be infinite when α is zero. The assumptions made in the simplification of the analytical model might lead to such a dependence. However, this result gives us an insight about how α should be adjusted for minimizing $p_{\mathrm{air}}$, which is required to detach the gripper from the object by overcoming $\sigma_{\mathrm{adh}}$. Accordingly, α should be selected as large as possible, but it should be feasible for real-life applications. A slope larger than 20 degrees is barely achievable in the authors’ opinion as it would make the fabrication of the gripper too difficult (the demolding process would rip the gripper apart). For this reason, 20 degrees is chosen as the maximum limit of α. This results in $p_{\mathrm{air}}/\sigma_{\mathrm{adh}}=7.55$ in theory.

## 3. Preliminary Experiments

In order to verify the analytical model from Equation (1), the gripping performance of soft bending actuators with different slopes is investigated.

### 3.1. Materials and Methods

Molds required for the fabrication of the soft bending actuators are produced by the 3D printer Form 2 (Formlabs). Gray Pro Resin is selected as the material of the molds because it provides a good surface quality and is less sticky to the cured elastomer. A release agent (Buehler) is also applied to the inner surfaces of the molds.

Each different slope requires a different mold couple consisting of an upper and a lower part. Five different angles are investigated experimentally: −20°, −10°, 0°, +10°, and +20°. Therefore, mold couples for each slope were produced.

Elastosil M 4601 (Wacker Chemie) is selected as the material for the gripper due to its several advantages in pneumatic applications. It is highly resistant to bending and elongation, and, in uncured form, it has a low viscosity which makes molding easier.

**Figure 6:**
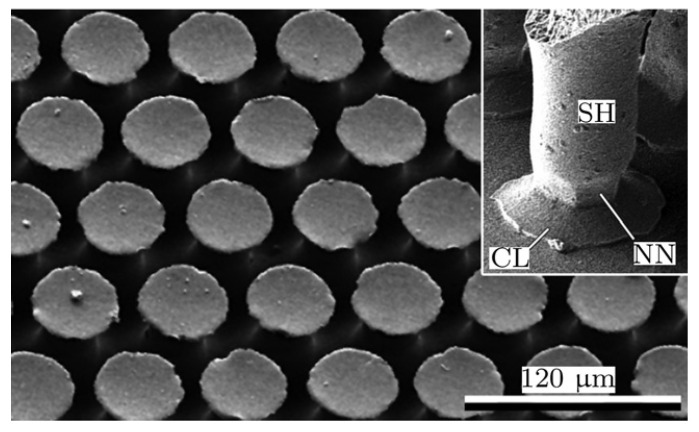
Scanning electron micrograph of mushroom-shaped adhesive microstructure (MSAMS). Inset shows an individual MSAMS in contact with smooth glass substrate. SH, shaft; CL, contact lip; NN, narrowed neck. Used with permission [18].

The commercial adhesive tape Gecko Nanoplast (Klettband Technik) made of silicone rubber is used for the adhesive layer. The adhesive structures are mushroom-shaped [8], and the tape has the following properties [16]: number of adhesive elements 29,000/mm${}^{2}$; tape thickness 0.35 mm; and weight 290 g/cm${}^{3}$. A scanning electron micrograph of the adhesive structures is shown in Figure 6.

Plexiglas (also known as acrylic glass) is used as gripping surface for the soft bending actuators and the gripper. It provides a smooth surface, which enhances adhesion, and is easy to clean.

Elastomeric prepregs [17] are used as the strain-limiting top layer of the gripper. In brief, they are textile semi-finished products that are preimpregnated with liquid (uncured) elastomer. Fleece (R&G Faserverbundwerkstoffe) is selected as textile material for easier impregnation with the elastomer. The fabrication process of a soft bending actuator using a prepreg as the strain-limiting layer is described in Appendix D. For each slope, three bending actuators are produced.

The bending actuator to be tested is carefully placed on a Plexiglas plate making sure that each air chamber is in contact with the surface. The air pressure inside the bending actuator is then increased by 0.01 bar every two seconds to fulfill the requirements of quasi-static balance and prevent any dynamic effects. Finally, the air pressure at which detachment of one of the outer air chambers takes place is recorded; this is defined as the detachment air pressure. These steps are repeated five times for each bending actuator. Before each experiment, the plate is cleaned to prevent systematic errors due to dust or dirt on the surface.

### 3.2. Results and Discussion

**Figure 7:**
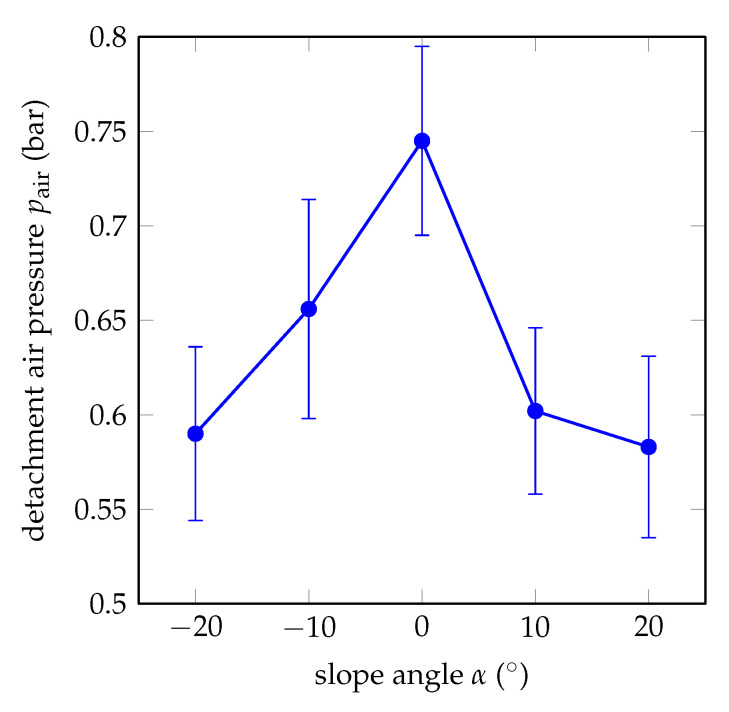
Experimental results of the detachment pressures for different slopes of soft bending actuators.

Figure 7 illustrates the experimental results of the detachment experiments. Here, circles represent the average values of the detachment pressures of three actuators and the vertical lines represent error bars. We can see that the highest detachment pressure is observed for α=0° with the average value of 0.74 bar. The lowest detachment pressures are observed for α=−20° and α=+20° with the average values of 0.58 bar and 0.59 bar, respectively. The bending actuators with α=−10° have their first detachment at 0.66 bar, and, similarly, the actuators with α=+10° have their first detachment at 0.61 bar. As the overall trend of the graph, the required pressure is maximal for α=0° and minimal for α=−20° and α=+20°, with a rough symmetry around α=0°. This is in accordance with the analytical model from Equation (5) and Figure 5. In the experimental results, the ratio of maximum to minimum air pressure is 1.32.

Figure 7 also includes the error bars of the measurements. There are many reasons for such high standard deviations. All actuators are fabricated hand-made and human-error cannot be prevented. For example, the mixing ratio of Elastosil M 4601 cannot be obtained very accurately, which affects the material properties of the elastomer. Another human error is introduced during the fabrication of prepregs; the amount of elastomer fleece is impregnated with cannot be adjusted very accurately and the elastomer cannot be applied homogeneously so that the strain-limiting layers of each actuator can be different in thickness and rigidity. Last but not least, although the surface of the Plexiglas is cleaned after every experiment, the adhesive tapes gather dust, which leads to a gradual decrease of the required detachment pressures through the five measurements of an actuator.

**Figure 8:**
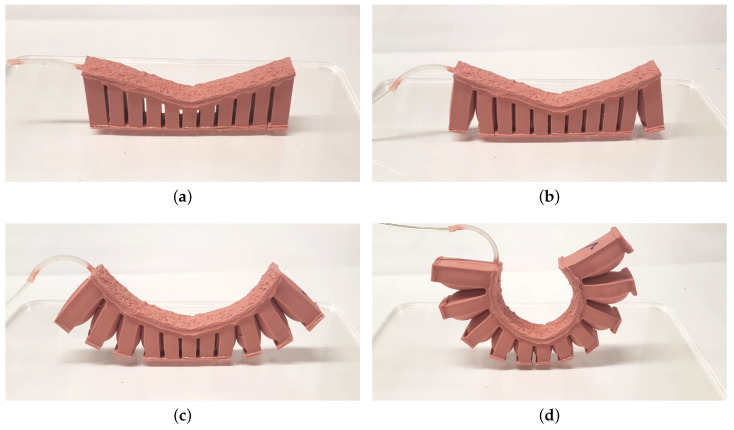
Sequential detachment of air chambers of an actuator with α=+20°: (**a**) attached, (**b**) most outer air chambers detached, (**c**) first three outer air chambers detached, (**d**) completely detached.

Figure 8 shows the sequential detachment process of the air chambers during air pressurization. Although the figure shows a symmetrical detachment behavior, some trials resulted in unsymmetrical detachment, which means that detachment of the two sides does not occur at the same time. However, this does not have any effect on the operation of the gripper since a complete detachment of all air chambers is required regardless of which one detaches first.

The analytical model and the experiments both show that actuators with higher slopes will detach easier, and this dependency is regardless of whether the slope is positive or negative. Thus, the same |α| dependency is assumed for the proposed gripper.

## 4. Embodiment Design

**Figure 9:**
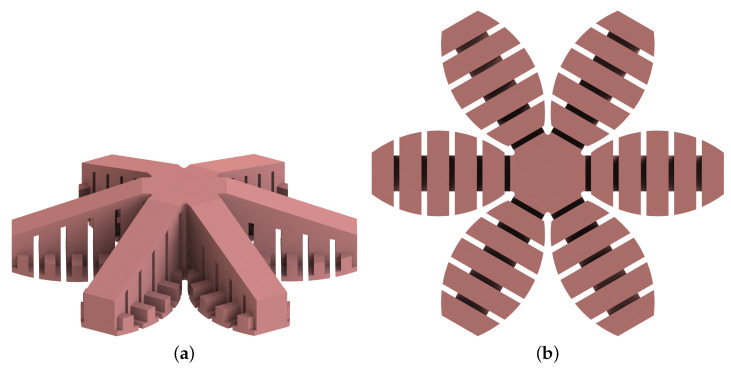
CAD model of the final design of the passive gripper with side extensions (α=−20°): (**a**) isometric view, (**b**) bottom view.

The embodiment design of the gripper is based on the foot of a gecko. Six actuator branches in star configuration are chosen for the final design to cover a large area. More branches would make the fabrication, and especially the demolding process, of the gripper more difficult. In order to increase the contact area, the gripper is extended by elliptical patches at the bottom of the gripper. The patches are reinforced by lateral ribs to ensure a certain stiffness. For minimizing the required air pressure, the individual toes of the gripper should be sloped. Both the analytical and experimental results show that the dependency of α is regardless of the sign and that the pressure is minimal at ±20°. For stability reasons, however, α=+20° is chosen since the entire mass is then concentrated in the center of the gripper. The CAD model of the final design of the gripper is illustrated in Figure 9. The minimum height of the gripper is 12.5 mm and the maximum height 30 mm. The distance between the ends of two opposite toes is 127 mm. Note that only the undersides of the patches are covered with Gecko Tape and the central hexagonal area is excluded as it is not actuated and thus does not contribute to the release process.

## 5. Gripper Performance

An experiment was conducted to estimate the maximum load weight that the passive gripper is able to carry. For this purpose, a cube made of Elastosil M 4601 with 3 cm${}^{2}$ side area was fabricated. The bottom face of the specimen was covered with Gecko Tape, filling the whole surface. Then, the specimen was attached to a Plexiglas plate. Using a rope, the specimen was pulled normally, while the detachment force was measured by a force gage.

The measured detachment force was 9 N. This value corresponds to 3 cm${}^{2}$ of the adhesive tape, whereas the total area of the bottom face of the passive gripper (except the central hexagonal area) is 60 cm${}^{2}$. Thus, the load capacity of the passive gripper is 180 N, which means that the passive gripper can (theoretically) carry a load with a weight of up to 18 kg. In comparison, the weight of the gripper is 120 grams.

In order to validate this theoretical result, we performed tensile tests with the gripper using a digital force gauge (Model FH 500, Sauter GmbH). In particular, the gripper was placed on a Plexiglas plate and then a force was applied at the center of the gripper using a rope. The force was continuously increased manually until the gripper detached from the plate. The result was a tensile force equivalent to about 5 kg, which is about 30% of the theoretical result. The reason for this deviation can be seen in the elastic deformation of the gripper as well as a slight contamination of the adhesive tape.

**Figure 10:**
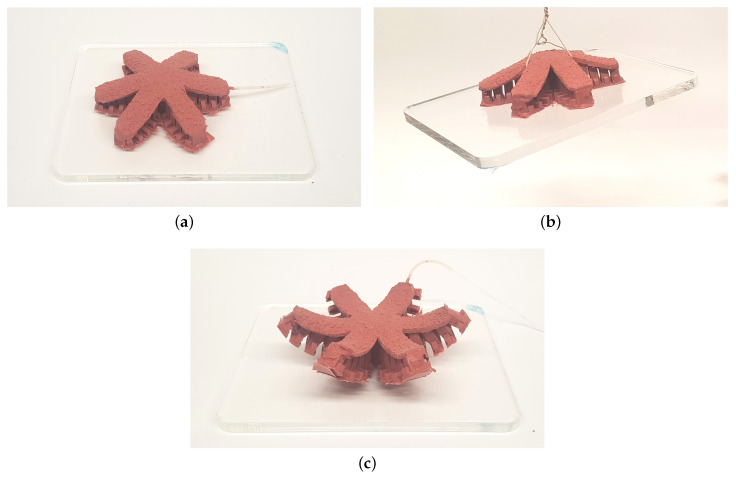
Operation of the passive gripper: (**a**) attached, (**b**) carrying, (**c**) detached.

Additionally, a full operation of the passive gripper was tested. Here, a Plexiglas plate with the weight of 480 g was selected as the load. The passive gripper becomes directly attached when it is put on the plate. Our own experiments showed that even the weight of the tape is enough for the tape to stick to the surface. Vacuuming can be applied for a short amount of time to further improve contact. Figure 10 shows the operation of the soft passive gripper. The gripper adheres to the plate without applying any force and can lift it without deforming greatly. The release process corresponds largely to that of a single actuator in Figure 8, where the outer chambers are first released and the release process of the chambers progresses inwards until the gripper is completely detached. The gripper is also fully functional under water as we have also tested the gripper by placing it in the center of a water-filled bowl and then lifting it, cf. also [19].

## 6. Conclusions

In this paper, a bio-inspired soft passive gripper which can attach on and carry flat objects was developed and analyzed. The passive adhesion ability is acquired via an adhesive tape. The gripper is made of Elastosil M 4601 and a prepreg with fleece fabric is used as the inextensible layer.

An analytical model based on Euler–Bernoulli beam theory suggests that, with an increasing slope angle of the actuator, the required detachment air pressure drastically decreases. For the validation of the analytical model, three actuators for each of the angles −20°, −10°, 0°, +10°, and +20° were fabricated and the corresponding detachment air pressures were measured. The experimental results supported the analytical results. The minimum detachment air pressure was observed at ±20° and the maximum detachment air pressure at 0°. Furthermore, the angles ±10° showed similar detachment air pressures, suggesting a symmetric shape. Finally, a case study of a passive gripper with six branches and a slope of +20° was presented. After fabrication, load-carry operations were conducted successfully.

Automated load carrying systems are important parts of today’s industry. Soft robotics is also becoming an essential part of the future’s technology. The presented gripper brings these two things together and shows successful results in load-carrying operation tests. First of all, the developed soft gripper requires no initial force to attach to a flat surface, and, more importantly, the gripper does not require any additional energy to sustain its attachment during the carrying operation. These properties make this soft gripper a possible substitute to already existing automatic load carrier systems such as mass crane and hoist. In addition, the analysis of sloped actuator designs showed promising results in terms of gripper geometry optimization. Even though using a sloped design showed great improvements for passive grippers, this design can also show great benefits for active soft grippers. For the same air pressure, the sloped design would provide higher forces at the tip of the actuator than with flat actuators. This would result in a firmer gripping performance.

## Figures and Tables

**Figure A1:**
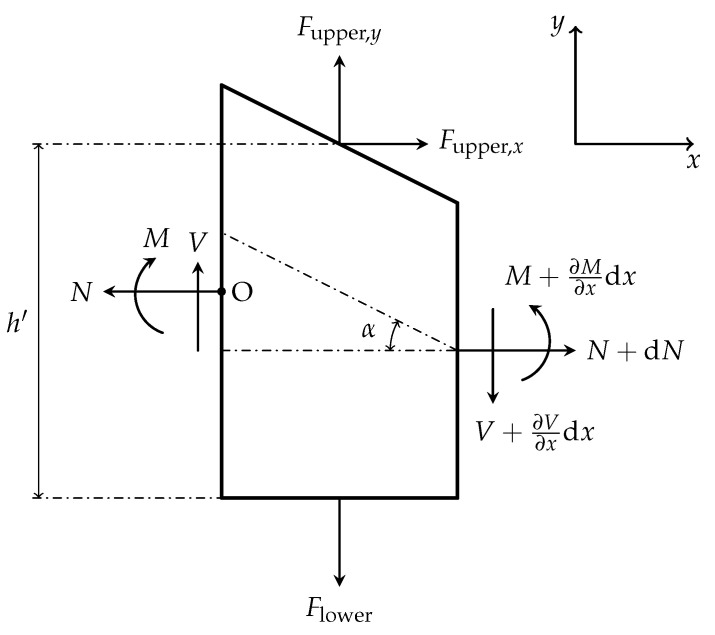
Free-body diagram of the infinitesimal element.

**Figure A2:**
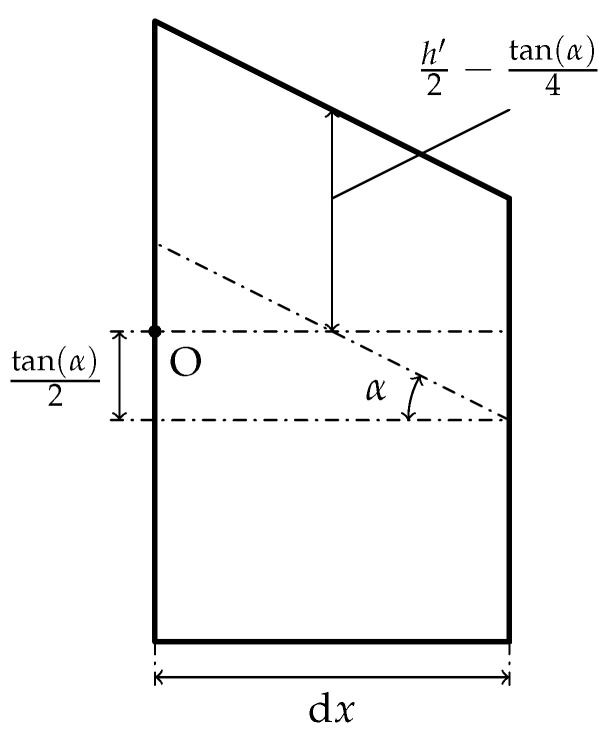
Moment arms of the forces.

**Figure A3:**
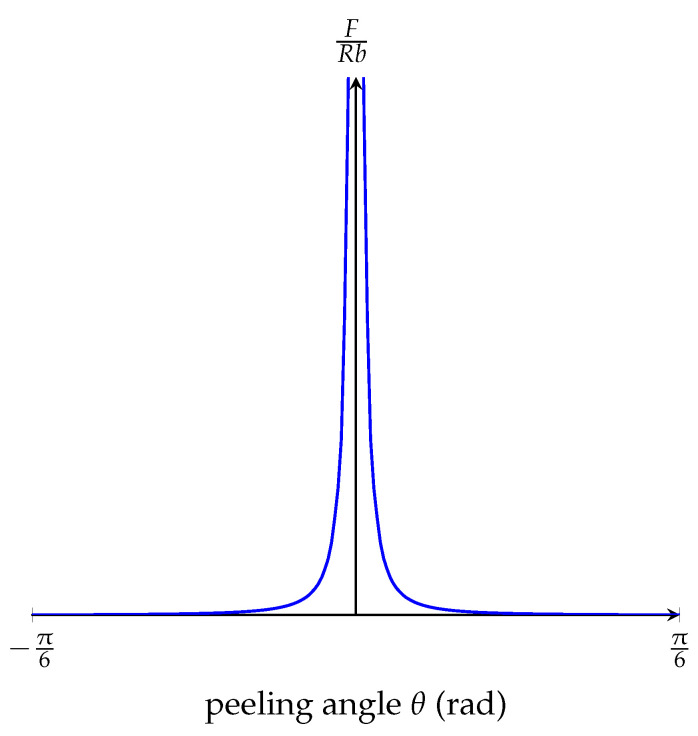
Graph of Equation (A30).

**Figure A4:**
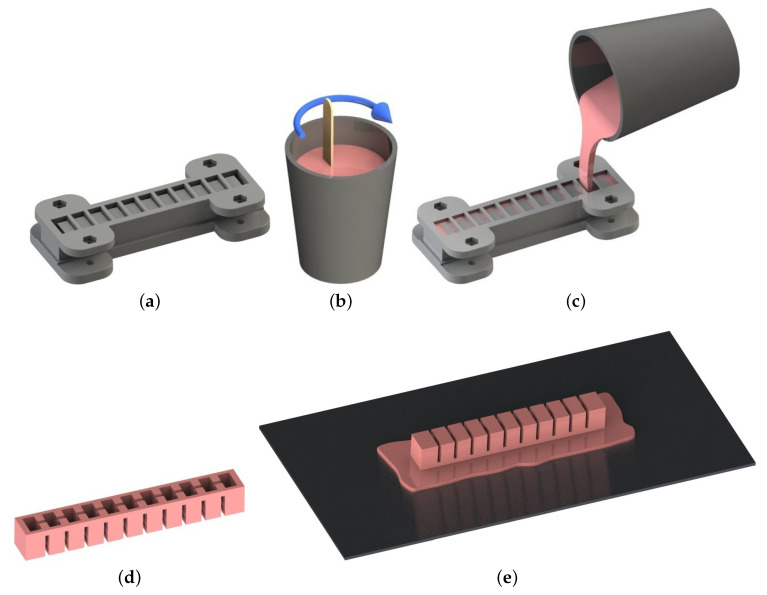
Fabrication steps of a soft bending actuator with a prepreg as strain-limiting layer (α=0°): (**a**) assembly of the mold, (**b**) mixing of elastomer, (**c**) filling the mold, (**d**) extracting the cured part from the mold, (**e**) placing the cured part on the prepreg layer.

## Appendix A. Static Equilibrium of Detachment

In order to determine a relationship between the air pressure $p_{\mathrm{air}}$ needed to detach the gripper from a flat object and the adhesive stress $\sigma_{\mathrm{adh}}$ applied by the adhesive layer, a static equilibrium is assumed where the toe structure, which is represented by a bending actuator, is attached to the object and does not have any transverse deformation. An infinitesimal thin layer of length d*x* is vertically cut from an air chamber of the sloped bending actuator (cf. Figure 4b) and the interior of the chamber is assumed to consist of solid material with a constant Young’s modulus. The mechanical influence of the non-stretchable layer is neglected here.

The free-body diagram of the infinitesimal element is depicted in Figure A1. The air pressure acts on both the upper and lower layer, whereby, due to the slope α, the upper force is divided into components in the *x* and *y* direction (*t* is the thickness):

(A1) $$\begin{array}{cc} F_{\mathrm{upper},x} & =p_{\mathrm{air}}\tan\left(\alpha \right)\;t\;dx, \end{array}$$

(A2) $$\begin{array}{cc} F_{\mathrm{upper},y} & =p_{\mathrm{air}}\;t\;dx, \end{array}$$

(A3) $$\begin{array}{cc} F_{\mathrm{lower}} & =(p_{\mathrm{air}}+\sigma_{\mathrm{adh}})\;t\;dx. \end{array}$$

In addition to these three external forces, the bending moment *M*, the shear force *V*, and the normal force *N* act as internal forces. The equilibrium of forces in horizontal direction is given by

(A4) $$-N+N+dN+F_{\mathrm{upper},x}=0.$$

Inserting Equation (A1) into Equation (A4) results in

(A5) $$\frac{dN}{dx}=-p_{\mathrm{air}}\tan\left(\alpha \right)\;t.$$

The equilibrium of forces in vertical direction becomes

(A6) $$F_{\mathrm{upper},y}+V-F_{\mathrm{lower}}-V-\frac{\partial V}{\partial x}dx=0,$$

which is simplified to

(A7) $$\frac{\partial V}{\partial x}dx=F_{\mathrm{upper},y}-F_{\mathrm{lower}}.$$

Inserting Equations (A2) and (A3) into Equation (A7) gives us

(A8) $$\frac{\partial V}{\partial x}dx=-\sigma_{\mathrm{adh}}\;t\;dx.$$

Applying the moment arms from Figure A2, the equilibrium of moments at point O becomes

(A9) $$\begin{array}{cc} 0 & =M-M-\frac{\partial M}{\partial x}dx-\left(N+dN\right)\frac{\tan(\alpha )}{2}dx+\left(V+\frac{\partial V}{\partial x}dx\right)dx\\ \; & \;+F_{\mathrm{lower}}\frac{dx}{2}-F_{\mathrm{upper},y}\frac{dx}{2}+F_{\mathrm{upper},x}\;\left(\frac{h^{\prime }}{2}-\frac{\tan(\alpha )}{4}dx\right). \end{array}$$

From classical beam theory [20], the bending moment *M* can be expressed in terms of the vertical deflection *w*:

(A10) $$M=-\frac{EI}{2}\frac{\partial^{2}w}{\partial x^{2}}.$$

Using this expression, the term $\frac{\partial M}{\partial x}dx$ in Equation (A9) can be calculated as follows:

(A11) $$\frac{\partial M}{\partial x}dx=-\frac{EI}{2}\frac{\partial^{3}w}{\partial x^{3}}dx.$$

However, this term vanishes as the vertical deflection of our infinitesimal element is neglected. In addition, the flexural rigidity EI of elastomers is very small so that it can also be assumed to be zero. Consequently, rewriting Equation (A9) with the above assumptions gives

(A12) $$0=-\left(N+dN\right)\frac{\tan(\alpha )}{2}dx+\left(V+\frac{\partial V}{\partial x}dx\right)dx+\frac{F_{\mathrm{lower}}}{2}dx-\frac{F_{\mathrm{upper},y}}{2}dx+\frac{F_{\mathrm{upper},x}}{2}\left(h^{\prime }-\frac{\tan(\alpha )}{2}dx\right).$$

Dividing each side by d*x* and multiplying by 2 results in

(A13) $$0=-\left(N+dN\right)\;\tan\left(\alpha \right)+2\;\left(V+\frac{\partial V}{\partial x}dx\right)+F_{\mathrm{lower}}-F_{\mathrm{upper},y}+F_{\mathrm{upper},x}\;\left(\frac{h^{\prime }}{dx}-\frac{\tan(\alpha )}{2}\right).$$

Bringing $\frac{\partial V}{\partial x}dx$ to the left-hand side and inserting Equation (A7) leads to

(A14) $$2\;\left(V+F_{\mathrm{upper},y}-F_{\mathrm{lower}}\right)=\left(N+dN\right)\;\tan\left(\alpha \right)-F_{\mathrm{lower}}+F_{\mathrm{upper},y}-F_{\mathrm{upper},x}\;\left(\frac{h^{\prime }}{dx}-\frac{\tan(\alpha )}{2}\right),$$

which simplifies to

(A15) $$2\;V=\left(N+dN\right)\;\tan\left(\alpha \right)+F_{\mathrm{lower}}-F_{\mathrm{upper},y}-F_{\mathrm{upper},x}\;\left(\frac{h^{\prime }}{dx}-\frac{\tan(\alpha )}{2}\right).$$

Using Equation (A5), d*N* can be rewritten as

(A16) $$dN=-p_{\mathrm{air}}\tan\left(\alpha \right)\;t\;dx.$$

Inserting Equations (A1)–(A3) and (A16), Equation (A15) becomes

(A17) $$2\;V=\left(N-p_{\mathrm{air}}\tan\left(\alpha \right)\;t\;dx\right)\;\tan\left(\alpha \right)+p_{\mathrm{air}}\;t\;dx+\sigma_{\mathrm{adh}}\;t\;dx-p_{\mathrm{air}}\;t\;dx-p_{\mathrm{air}}\tan\left(\alpha \right)\;t\;dx\;\left(\frac{h^{\prime }}{dx}-\frac{\tan(\alpha )}{2}\right).$$

Rearranging this equation results in

(A18) $$2\;V=N\tan\left(\alpha \right)-p_{\mathrm{air}}\tan^{2}\left(\alpha \right)\;t\;dx+\sigma_{\mathrm{adh}}\;t\;dx-p_{\mathrm{air}}\tan\left(\alpha \right)\;t\;h^{\prime }+\frac{1}{2}p_{\mathrm{air}}\tan^{2}\left(\alpha \right)\;t\;dx,$$

which is simplified to

(A19) $$2\;V=N\tan\left(\alpha \right)-\frac{1}{2}p_{\mathrm{air}}\tan^{2}\left(\alpha \right)\;t\;dx+\sigma_{\mathrm{adh}}\;t\;dx-p_{\mathrm{air}}\tan\left(\alpha \right)\;t\;h^{\prime }.$$

Taking the derivative of Equation (A19):

(A20) $$2\;\frac{dV}{dx}=\frac{dN}{dx}\tan\left(\alpha \right)-\frac{d}{dx}\left(\frac{1}{2}p_{\mathrm{air}}\tan^{2}\left(\alpha \right)\;t\;dx\right)+\frac{d}{dx}\left(\sigma_{\mathrm{adh}}\;t\;dx\right)-p_{\mathrm{air}}\tan\left(\alpha \right)\;t\;\frac{dh^{\prime }}{dx}$$

and introducing

(A21) $$\frac{dh^{\prime }}{dx}=\tan\left(\alpha \right)$$

leads to

(A22) $$2\;\frac{dV}{dx}=\frac{dN}{dx}\tan\left(\alpha \right)-\frac{d}{dx}\left(\frac{1}{2}p_{\mathrm{air}}\tan^{2}\left(\alpha \right)\;t\;dx\right)+\frac{d}{dx}\left(\sigma_{\mathrm{adh}}\;t\;dx\right)-p_{\mathrm{air}}\tan^{2}\left(\alpha \right)\;t.$$

Simplifying Equation (A8):

(A23) $$\frac{dV}{dx}=-\sigma_{\mathrm{adh}}\;t$$

and inserting Equations (A5) and (A23) results in

(A24) $$-2\;\sigma_{\mathrm{adh}}\;t=-p_{\mathrm{air}}\tan^{2}\left(\alpha \right)\;t-\frac{d}{dx}\left(\frac{1}{2}\;p_{\mathrm{air}}\tan^{2}\left(\alpha \right)\;t\;dx\right)+\frac{d}{dx}\left(\sigma_{\mathrm{adh}}\;t\;dx\right)-p_{\mathrm{air}}\tan^{2}\left(\alpha \right)\;t,$$

which, in turn, can be simplified as follows:

(A25) $$2\;p_{\mathrm{air}}\tan^{2}\left(\alpha \right)-2\;\sigma_{\mathrm{adh}}=-d\;\left(\frac{1}{2}\;p_{\mathrm{air}}\tan^{2}\left(\alpha \right)\right)+d\;\sigma_{\mathrm{adh}}.$$

By neglecting the infinitesimal terms on the right-hand side of Equation (A25), the relationship between $p_{\mathrm{air}}$ and $\sigma_{\mathrm{adh}}$ finally becomes

(A26) $$2\;p_{\mathrm{air}}\tan^{2}\left(\alpha \right)-2\;\sigma_{\mathrm{adh}}\approx 0,$$

which is simplified to

(A27) $$\frac{p_{\mathrm{air}}}{\sigma_{\mathrm{adh}}}\approx \frac{1}{\tan^{2}\left(\alpha \right)}=\cot^{2}\left(\alpha \right).$$

## Appendix B. Adhesive Tape Peeling Model

The dynamics of adhesive tape peeling is an important topic for modeling the passive gripper as the air pressure required for detachment is directly related to it. The Kendall model [15] is a typical adhesive tape peeling model. It describes the force required to overcome adhesion, which results in detachment of the tape. For a distance change of Δc during the peeling process, the energy of the surface changes as −bRΔc. Here, *R* is the adhesive energy, which is the experimental energy required to fracture a unit area of the interface, and *b* is the width of the tape. There is also a potential energy term $F\left(1-\cos(\theta )\right)\Delta c$, which results from the movement of the applied force *F*, and an elastic term due to the tape extension in the applied force’s direction (θ is the peeling angle). All these terms lead to

(A28) $${\left(\frac{F}{b}\right)}^{2}\frac{1}{2dE}+\frac{F}{b}\left(1-\cos(\theta )\right)=R,$$

where *d* is the thickness of the tape and *E* is the Young’s modulus of the tape material. Since in our case the elasticity of the tape is constrained by the adhesion of the gripper, the above equation becomes

(A29) $$\frac{F}{b}\left(1-\cos(\theta )\right)=R,$$

which can be rewritten as

(A30) $$\frac{F}{Rb}=\frac{1}{1-\cos(\theta )}.$$

This equation describes the ratio between the applied force and the adhesion in terms of the peeling angle. As depicted in Figure A3, the applied force required for overcoming adhesion steeply decreases as the peeling angle increases, which is an important factor for optimizing the passive gripper. The peeling angle should be increased for minimizing the required force applied to the adhesive tape. However, the Kendall model is based on the peeling of an elastic tape and may not be accurate for the peeling of an entire actuator.

## Appendix C. Analogy between Static Equilibrium Model and Kendall Model

When comparing Figure 5 to Figure A3, a similarity between the static equilibrium model and the Kendall model is observed, since they both go to infinity as |α|, respectively |θ|, approaches zero and vanish as |α|, respectively |θ|, takes larger values. The reason for this is that Equations (A27) and (A30) are strongly related to each other, considering that they both express the relationship between the applied force and adhesion. In the Kendall model, *F* is a directly applied force pulling the adhesive tape off the surface, and, in the static equilibrium model, the resultant force is due to the air pressure inside the chambers. The *R* term in Equation (A30) is the energy required for fracture of adhesive microstructures, and it is directly related to $\sigma_{\mathrm{adh}}$. However, the angles θ and α are not the same geometric parameter; θ is the detachment angle of the adhesive tape and α is the slope of the toe structure design. The geometric relationship between θ and α can be investigated by comparing Equations (A27) and (A30). The right-hand side of Equation (A30) gives us the ratio between the applied force and the adhesion stress and is therefore linearly proportional to the ratio between $p_{\mathrm{air}}$ and $\sigma_{\mathrm{adh}}$:

(A31) $$\frac{1}{1-\cos(\theta )}\propto \cot^{2}\left(\alpha \right),$$

(A32) $$1-\cos\left(\theta \right)\propto \tan^{2}\left(\alpha \right).$$

θ can be explicitly expressed as

(A33) $$\theta \propto \cos^{-1}\left(1-\tan^{2}\left(\alpha \right)\right).$$

By adding a proportionality constant *k*, θ can be estimated as

(A34) $$\theta =k\cos^{-1}\left(1-\tan^{2}\left(\alpha \right)\right).$$

This approximation is valid only for the initial detachment as the slope of the toe structure changes significantly during bending. The constant *k* can be estimated experimentally.

As a result, the analogy between the Kendall model and the static equilibrium model describes how the detachment angle of the adhesive tape is related to the slope of the toe structure, and, therefore, helps us to understand the correlation between the kinetics of the soft bending actuator and the kinetics of adhesive tape peeling.

## Appendix D. Fabrication of Actuators and Gripper

Figure A4 illustrates the steps of fabrication of soft bending actuators with α=0°. At first, molds for the corresponding slope are manufactured via additive manufacturing and then assembled, (a). In order to prevent any elastomer leakage from the mold assembly, the two mold parts are fixed by bolted connections. The elastomer is mixed, (b), and then kept in a vacuum chamber for five minutes to remove air bubbles inside the mixture. The elastomer mixture is poured into the mold assembly, (c), and then cured in an oven at 60 °C for 45 min. After curing, the part is extracted from the mold, (d). For adding the strain limiting layer, a piece of fabric (fleece) is impregnated with uncured elastomer on a plate using a roller, and then the already cured bottom part of the actuator is placed on it, (e). After further curing the whole actuator, excess prepreg is cut away and the actuator is ready to use.

After fabrication of the main body, a plastic tube is inserted into the bending actuator and sealed with uncured elastomer. Additionally, as the last step, gecko tape is glued to the bottom surface of the actuator with uncured elastomer, and then the actuator is finally cured in an oven at 60 °C for 20 min (assuming that the gecko nanostructures are not destroyed at such temperature).

The fabrication steps are the same for the bending actuators with other α angles except the step of the strain limiting layer. Here, a piece of fabric, which is impregnated with uncured elastomer, is placed on top of the cured part extracted from the mold couple.

The gripper is fabricated in the same way as the bending actuators but with different molds. Here, the mold couples are also fastened by bolted connections to prevent elastomer leakage.
